# Visualizing dynamics of angiogenic sprouting from a three-dimensional microvasculature model using stage-top optical coherence tomography

**DOI:** 10.1038/srep42426

**Published:** 2017-02-10

**Authors:** Haruko Takahashi, Keisuke Kato, Kenji Ueyama, Masayoshi Kobayashi, Gunwoong Baik, Yasuhiro Yukawa, Jun-ichi Suehiro, Yukiko T. Matsunaga

**Affiliations:** 1 Center for International Research on Integrative Biomedical Systems, Institute of Industrial Science, The University of Tokyo, 4-6-1 Komaba, Meguro-ku, Tokyo 153-8505, Japan; 2 R&D Department 1, Screen Holdings Co., Ltd., 322 Furukawa-cho, Hazukashi, Fushimi-ku, Kyoto 612-8486, Japan; 3 Center for Interdisciplinary Research on Micro-Nano Methods, Institute of Industrial Science, The University of Tokyo, 4-6-1 Komaba, Meguro-ku, Tokyo 153-8505, Japan; 4 Department of Pharmacology and Toxicology, Kyorin University School of Medicine, 6-20-2 Shinkawa, Mitaka-shi, Tokyo 181-8611, Japan

## Abstract

Three-dimensional (3D) *in vitro* microvasculature in a polydimethylsiloxane-based microdevice was developed as a physiologically relevant model of angiogenesis. The angiogenic process is monitored using stage-top optical coherence tomography (OCT). OCT allows non-invasive monitoring of the 3D structures of the prepared host microvasculature and sprouted neovasculature without fluorescence staining. OCT monitoring takes only a few minutes to scan through the several-millimetre scale range, which provides the advantage of rapid observation of living samples. The obtained OCT cross-sectional images capture 3D features of the angiogenic sprouting process and provide information on the dynamics of luminal formation. The stage-top system used in this study enables the observer to visualize the *in vitro* dynamics of 3D cultured cells simply and conveniently, offering an alternative monitoring method for studies on angiogenesis and providing quantitative information about vascular morphological changes.

Angiogenesis is the physiological process for *de novo* vascular network formation from pre-existing blood vessels^1^,^2^,^3^. It is often accompanied by lumen formation and supplies oxygenated and nutrient-rich blood to avascular tissues in the body, which is vital for human development. In contrast, abnormal angiogenesis, characterized by immature, leaky and heterogeneous vascular phenotypes, is associated with the prognosis of many types of diseases, such as inflammation, diabetic retinopathy and tumor growth^4^. It is therefore an attractive drug target and various anti-angiogenic agents are already on the market. Recently, a large number of studies have shown that anti-angiogenic therapies transiently normalize abnormal vasculatures and vascular remodeling can alter the efficacy of combined chemotherapy^5^,^6^. While the emerging concept has provided new insight for pharmacological strategies of anti-angiogenic therapies, the underlying mechanism remains unclear because the methods for microscopic observation are limited in spatial-temporal resolution. If it is capable to evaluate the structural change of vasculatures, especially of sprouting points, responding to anti-angiogenic drug with spatial information in real time, pharmacological target sites and cellular/tissue responses can be investigated in more details. Thus, there is a growing need for non-invasive and real-time monitoring systems for capturing architectural and structural change of vessels accompanying anti-angiogenic treatment.

To date, angiogenesis has been evaluated using several valuation points, including the number of sprouts, length of the sprouts, and vascular density. Angiogenesis has been studied *in vitro* since the 1980 s^7^,^8^. Initial reports described vasculature-like lumen formation from endothelial cells on extracellular matrices. Later, the generation and elongation of sprouts attracted attention. These findings prompted the development of sprouting models from embedded cells on micro-beads, the ‘beads assay method’, and isolated vessels from the body, the ‘*ex vivo* method’^8^. In some cases, however, new vasculature from these models included primitive sprouts without lumens. On the other hand, recent advances in tissue engineering combined with microfabrication and microfluidics have enabled the development of microengineered *in vitro* models of the functional tissues. Several *in vitro* angiogenesis models in silicone-based microdevice were reported^9^,^10^. These devices enable to monitor angiogenic sprouting morphogenesis under the microscope.

Identifying matured functional sprouts with lumen formation and detecting the lumen-forming process are essential for precise observation of angiogenesis. Information on geometrical or structural properties is important for detecting lumen formation, but to date there is no simple, versatile method for monitoring dynamic changes of cells and tissues. In addition, it would be a significant advantage to be able to see dynamic changes in an entire vasculature as well as the developing neovasculature over time to detect physiological phenomena during the angiogenic process. The blood vessels in the body appear in a variety of sizes, with diameters ranging from ten to hundreds of microns. Thus, wide-range and longitudinal imaging of both the host (parent) vasculature and the neovasculature is required for these investigations.

There have been substantial efforts to visualize cells under precise, appropriate conditions^11^. Traditionally, brightfield microscopy (BFM) has been utilized for imaging live cells. The images obtained, however, are two-dimensional (2D) and planar and therefore are inappropriate for observing three-dimensional (3D) lumen formation. Confocal laser scanning microscopy (CLSM) allows 3D reconstruction of images but requires fluorescence staining of target molecules, cells, and tissues, which limits long-term live imaging. Additionally, high intensity laser irradiation is phototoxic against fluorescent probes, which causes fluorescence photobleaching. CLSM microscopes also are weak in regard to high resolution when scanning along the optical (z) axis, which is responsible for inaccurate measurements of samples across an area of thickness. Recently, light sheet fluorescence microscopy (LSFM) has been developed and has better multi-angle and less phototoxic observations^12^. The LSFM has several advantages over CLSM, including high-speed scanning, reduced phototoxicity from fluorescence, and deep imaging through thick tissues. It is not a versatile method at this point, however, because of the unique specimen preparation required: A transparent sample embedded in a cylindrical gel is needed, complicating observation of a sample in a cell culture dish. Optical coherence tomography (OCT) has been developed for non-invasive, cross-sectional imaging in biological systems^13^. It is already known as a versatile imaging method in the ophthalmological field, especially for funduscopic examination of the retina^14^. OCT uses low-coherence interferometry to produce a 2D cross-sectional image of optical scattering from internal tissue microstructures. 3D images can then be constructed from these 2D images. OCT also allows imaging samples with a wide range of thickness (<2 mm). OCT techniques, however, are unorthodox in the area of research and development because there are few accessible systems with stage-type implementation^15^ suitable for microplates and cell culture dishes.

In this work, we evaluated stage-top modelled OCT as a convenient alternative method for 3D live imaging of vessel structure change during angiogenesis *in vitro*. We evaluated dynamic angiogenic sprouting from prepared 3D microvasculatures in a polydimethylsiloxane (PDMS)-based microdevice, with a focus on live monitoring of neovasculature elongation and lumen formation. We found that OCT is an ideal method that allows (i) non-invasive monitoring, (ii) quick scanning in a wide range and depth, and (iii) 3D visualization of vessels’ luminal structures and quantitative analysis of structural parameters. This report describes the use of OCT techniques for detecting the dynamics of *in vitro* cultured cells to provide a new avenue for studying angiogenesis.

## Results and Discussion

### 3D *In Vitro* Angiogenesis Model with the PDMS Microdevice

**Figure 1:**
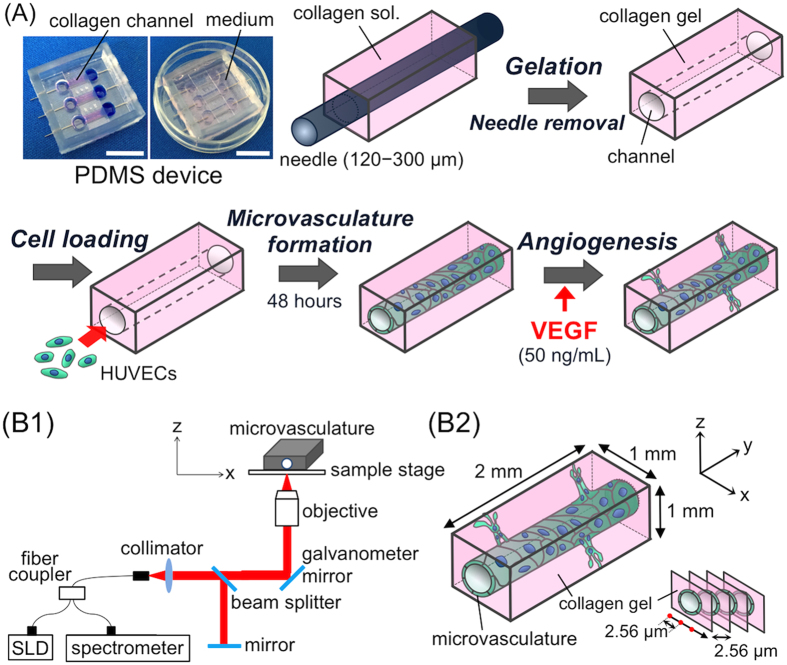
Concept presented in this study. (**A**) Polydimethylsiloxane (PDMS)-based microdevice and 3D microvasculature for *in vitro* angiogenesis model. Bar = 1.0 cm. (**B1**) Stage-top optical coherence tomography (OCT) setup. The super luminescent diode (SLD) output is coupled into a single mode fibre and split at the fibre coupler into the microvasculature sample and reference arms. Reflections from the two arms are combined at the coupler and detected by the spectrometer. (**B2**) Scanning condition for the microvasculatures. Longitudinal imaging was performed in the area of microvasculature: where x = 1 mm, y = 2 mm, z = 1 mm. VEGF, vascular endothelial growth factor.

For the *in vitro* angiogenesis model, we prepared a microvasculature with endothelial cells using the bottom-up approach via a needle method^9^,^10^. This method can provide a functional and perfusable 3D microvasculature model which grows and develops new vessel-like structures in response to inflammatory stimuli, reflecting the *in vivo* angiogenic process. In addition, the model captures dynamic changes of the entire vasculature, including both angiogenic sprouting and alterations in the host/parent microvasculature. As the dynamics of the host microvasculature is also significant throughout the angiogenic process, microvessels of several sizes (120, 200, and 300 μm in diameter) were monitored. To that end, we designed a PDMS-based, three-channel microdevice and fabricated microvasculatures, as summarized in Fig. 1A.

The PDMS microdevice has three dumbbell-shaped microvasculature chambers with channels [outside diameter (O.D.) 300 μm] through which to insert needles. Human umbilical vein endothelial cells (HUVECs) were chosen as a representative endothelial cell source, and type I collagen was chosen as the extracellular matrix for microvasculature fabrication. Acupuncture needles (120, 200, and 300 μm in diameter) were inserted across the PDMS device, and collagen solution was added to fill the central rectangular portion of each chamber. After the collagen gelled via incubation for 30 min at 37 °C, the needles were removed to leave a tubular opening in the collagen gel. A HUVEC suspension was injected into the remaining micro-channel and cultured in the medium at 37 °C for 2 days to allow microvasculature formation. After the 2-day incubation, the diameter of the microvasculature and the cell density on the collagen walls were measured (Fig. S1 in the Supplementary Information). The diameters of the fabricated microvasculatures created with 120, 200, and 300 μm micro-needles were measured by BFM (Fig. S1A) and found to be 80.9, 180.9, and 293.8 μm, respectively. The sizes of microvasculature tended to shrink slightly (~33%) compared with the sizes of the prepared micro-channels. This shrinkage was likely due to the cell−substrate and cell−cell interactions performed on the relatively soft collagen walls. To determine the cell density of HUVECs on the walls of each micro-channel, the HUVECs’ nuclei were stained and viewed using CLSM. Figure S1B shows that the HUVECs adhered to collagen walls, forming a monolayer at densities of 1.6–1.8 × 10^5^ cells/cm^2^ over all sizes of microvasculature. This demonstrated that uniformly cell-adherent microvasculatures with various pre-set diameters were successfully prepared in the PDMS microdevice.

### Angiogenesis Induced by Vascular Endothelial Growth Factor

**Figure 2:**
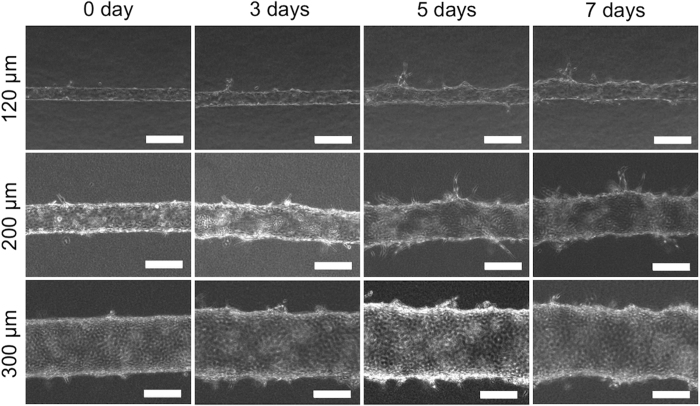
Bright field microscopy (BFM) images of angiogenesis process induced by VEGF (50 ng/mL). Bars = 200 μm.

To induce angiogenesis on prepared microvasculatures in the PDMS device, vascular endothelial growth factor (VEGF) (50 ng/mL) was added to the medium and incubated for 7 days. Changes in the microvasculature after addition of VEGF was monitored by BFM (shown in Fig. 2). VEGF is a growth factor that stimulates vasculogenesis and angiogenesis induced by macrophages, platelets, keratinocytes, and tumour cells^16^,^17^. A number of studies have induced angiogenesis by adding a range of VEGF concentrations^18^, finding that the sprouting of newer vessels from the microvasculatures was highly reproducible under the VEGF condition of 50 ng/mL in the same system that we used. Also, no sprouting of new vessels was detected in incubated microvasculatures without the VEGF addition (see Fig. S2 in the Supplementary Information), indicating that the sprouting behaviour was induced by the VEGF supplements. The diameters of the microvasculatures expanded over time, and their smooth, straight tubular structures changed into bumpy, bellows-like structures. Sprouting from prepared microvasculatures was observed beginning 2 days after VEGF stimulation, with the sprouts continuously elongating for up to 7 days. This sprouting action over time well agreed with that found in previous reports^7^,^18^. Interestingly, formation behaviour of neovascular vessels was characterized by the sizes of the prepared microvasculatures. Longer and larger neovascular vessels formed from host microvasculature with smaller diameters (O.D. 120 and 200 μm), while only small sprouts were detected from large microvasculatures (O.D. 300 μm). The cell number and density in each PDMS-device (approximately 1.5 × 10^6^ cells/device at initial seeding) was approximately the same in the three microvasculature models, suggesting the same oxygen state. But the effect of small diameter on hypoxic condition could not be ruled out. Another potential explanation is a geometrical cue rather than biochemical and biophysical cues surrounding the HUVECs. The role of diameter of host microvasculature in angiogenic sprouting process still remains unclear at this point and further investigation is needed in future work. Although the angiogenic sprouting from pre-formed 3D microvasculatures was observed live by BFM, lumen formation of neovascular vessels could not be seen using this method.

### Visualization of Angiogenesis

**Figure 3:**
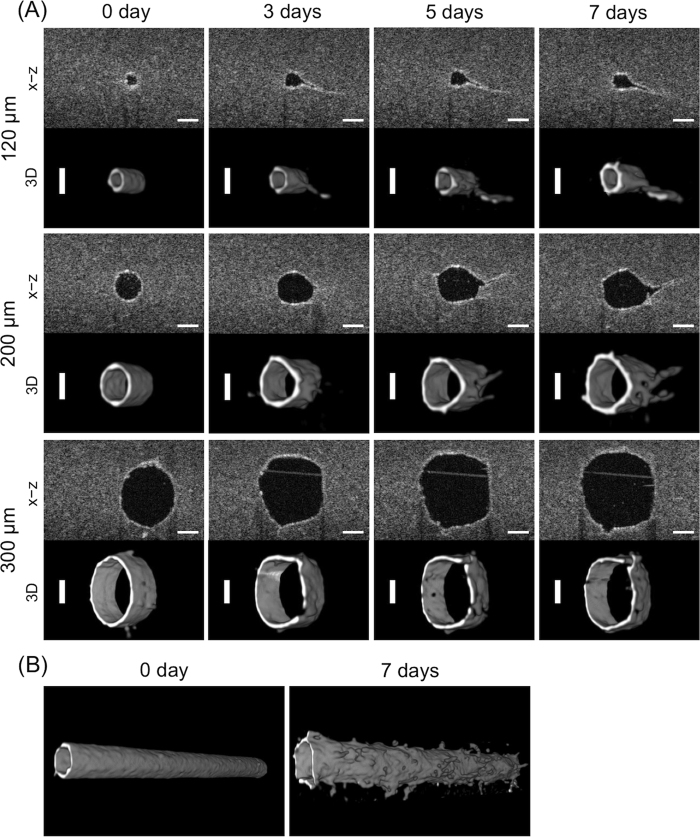
Optical coherence tomography (OCT) images of the angiogenic process induced by VEGF (50 ng/mL). (**A**) Partial time points of the microvasculatures’ sprouting. Top row: cross-sectional images surrounded by collagen gel. Bars = 100 μm. Bottom row: reconstructed 3D images performed using ImageJ software. Bars = 100 μm. (**B**) 3D images of the entire microvasculature (2 mm in length) with 200-μm diameter at days 0 and 7.

The change of microvasculatures after stimulation by VEGF was also monitored by OCT. Figure 1B describes the OCT setup used in this experiment. OCT provided cross-sectional images with a slice thickness of 2.56 μm, as shown in Fig. 3A. OCT permits obtaining live images non-invasively with no staining needed and high scan speed. For example, the observational time for one microvasculature in this study (scan area: x × y × z: 1 × 2 × 1 mm) was ~5 min. The obtained x−z plane cross-sectional images were reconstructed to 3D images using ImageJ software, as shown in Fig. 3A. OCT determined the increasing diameters of the microvasculature over time as well as BFM. Additionally, the luminal areas of the microvasculature were detected. It should be noted that the formation of the new vessel’s lumen was also visualized over time. Changes in the microvasculature were clearly indicated on reconstructed 3D images of the entire microvasculature as shown in Fig. 3B, which shows the shape changing to a bumpy, bellows-like tubular structure and outward sprouting of the neovasculature (also see movies in the Supplementary Information).

### Characterization of Neovasculature

**Figure 4:**
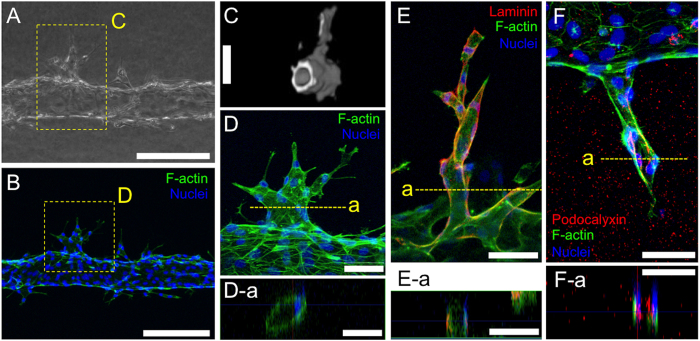
Characterization of neovasculature in the 3D *in vitro* angiogenesis model. (**A**) Brightfield microscopy (BFM) images. Bar = 200 μm. (**B**) Confocal laser scanning microscopy (CLSM) images. Bar = 200 μm. (**C**) Optical coherence tomography (OCT) images. The shown images corresponded to areas bounded by the yellow box in the BFM image. Bar = 100 μm. (**D**) Neovascurature stained for F-actin and nuclei. The shown images corresponded to areas bounded by the yellow box in the CLSM image. (D-a) In-plane section of neovascurature shown in (**D**). Bar = 50 μm. (**E**) Neovascurature stained for laminin. (E-a) In-plane section of neovascurature shown in (**E**). Bar = 50 μm. (**F**) Neovascurature stained for podocalyxin. (F-a) In-plane section of neovascurature shown in (**F**). Bar = 50 μm. All images are obtained 7 days after VEGF (50 ng/mL) stimulation. Host microvasculature O.D. = 120 μm.

In order to characterize the neovasculature from host microvasculature, we first compared images of neovasculature from BFM, CLSM, and OCT for observation of angiogenesis in our 3D *in vitro* models (Fig. 4A–C). As discussed above, BFM allows live imaging of cells but provides only 2D and planar images that do not allow evaluation of 3D vasculatures (Fig. 4A). OCT can provide both types of information: 3D structure and live imaging (Fig. 4C). However, resolution of OCT is 1–10 μm in x-, y- and z- axis directions, which are lower than that of optical microscope systems (200–1000 nm), limiting it to imaging details of cell structure. In contrast, CLSM allows observation of 3D structures with higher resolution (200–1000 nm in x- and y- axis and 500−1000 nm in z-axis directions), but requires fluorescence staining. To see the microvasculature under CLSM, the microvasculatures must undergo a 7-day incubation period, after which they are fixed. The actin fibers and nuclei are then stained using fluorescence-labelled phalloidin and Hoechst 33342, respectively (Fig. 4B and D). On CLSM images, actin fibers in the HUVECs were clearly shown. Also, the luminal structures of the neovasculature that continued from existing microvasculature were confirmed (Fig. 4D-a), but these images could only be obtained at the end-point of the experiment, thereby lacking ongoing assessment over time. To evaluate if organized neovasculature formation occurred in our 3D *in vitro* model, apical-basal polarity was investigated using immunofluorescence staining on 7-day cultured microvasculatures (Fig. 4E and F). For basolateral side, laminin deposition was observed. As shown in Fig. 4E and cross-sectional image (Fig. 4E-a), laminin was deposited outside of the luminal structure where cells contact the collagen gel channel. On the other hand, sialoglycoprotein podocalyxin as an apical marker localized around the luminal space in the neovasculature (Fig. 4F and F-a). These results were in good agreement with previous study^9^ and suggested that the sprouted neovasculatures from host microvascularture had matured luminal structures with physiologically relevant structural features, apical-basal polarity.

### Neovascular Formation from Prepared Microvasculature

**Figure 5:**
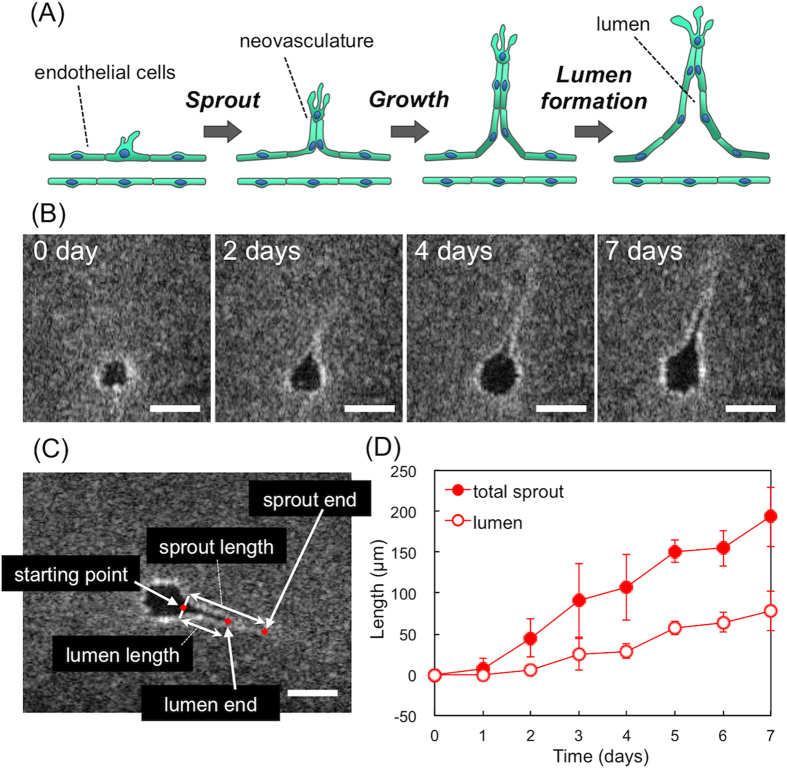
Analysis of sprouts from the prepared host microvasculature. (**A**) Vascular sprouting model^19^,^20^,^21^. (**B**) Neovascular formation detected by OCT. Host microvasculature O.D. = 120 μm, VEGF (50 ng/mL). Bar = 100 μm. (**C**) Measuring analysis of the sprouted neovasculature. (**D**) Sprouting kinetics of the neovasculature. Filled circles represent the total sprout length. Open circles represent the lumen length. Host microvasculature O.D. = 120 μm, VEGF (50 ng/mL). Bar = 100 μm.

The dynamic changes of angiogenesis over time were analysed using cross-sectional images obtained from OCT, which are summarized in Fig. 5. Several parameters (e.g., number and length of sprouts) have been used to evaluate angiogenesis using BFM and CLSM^7^,^8^,^18^, but these images lack information on the luminal structure. We focused on viewing the entire growth period, until the sprouts had gained their total length and we could visualize the lumens of the neovasculature. The vascular sprouting mechanism in the angiogenic process has been studied by a number of laboratories, and a model (called ‘cord hollowing model’) is offered (Fig. 5A)^19^,^20^,^21^. The process of luminal formation, however, is still under debate because direct, live observation is challenging.

Figure 5B shows the typical angiogenic process for prepared microvasculature. In OCT cross-sectional images detecting the sprouting area, a precise circular vasculature was observed initially (day 0). OCT detected a difference in light interference. The region of cells were shown as a strong white signal surrounded by a grey background, which came from collagen scattering. The lumen was shown as black. Two days after incubation with VEGF (50 ng/mL), the shape of the vasculature was dynamically changed, with a neovasculature sprout arising from the host microvasculature. The sprout continued to grow for the next 7 days (details shown in Fig. 5C). We then manually established the starting points to the sprout and luminal end points in each cross-sectional image. The total sprout length between the starting point and end point, and the luminal length between the starting point and end point were measured. Figure 5D shows the sprouting kinetics of the neovasculature. A void area (lumen) of the sprouted neovasculature was smaller than the total sprout, indicating that the endothelial cells that constructed the neovasculature were initially attached at their tips and then parted to form the mature lumen. These results could not provide a conclusive molecular mechanism in the vascular sprouting process directly, however it is demonstrated that the OCT images captured a structural relevance to the debated the cord hollowing model^19^,^20^,^21^.

### Dynamics of the Entire Microvasculature

**Figure 6:**
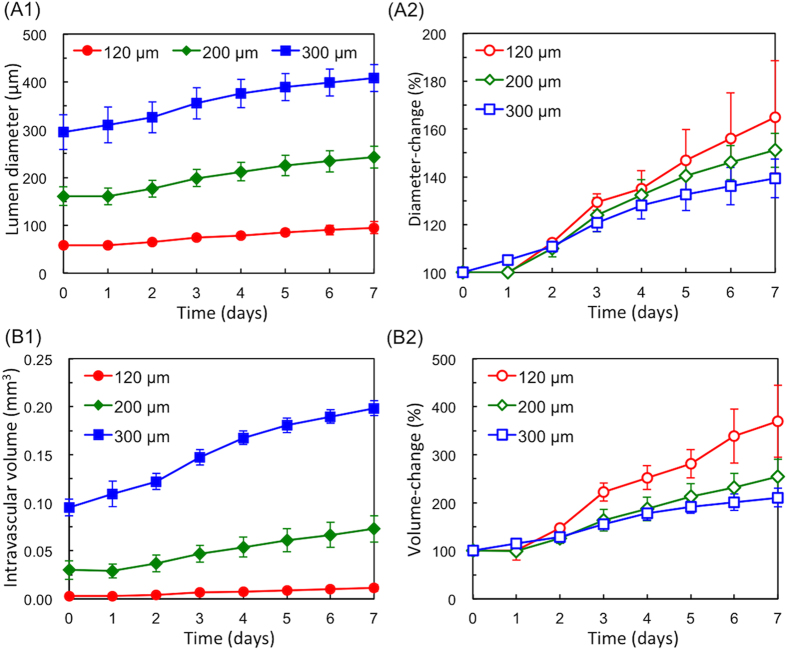
Dynamic changes in the entire microvasculature as analysed on OCT images. (**A**) Analysis results of the lumen diameter of the prepared host microvasculature: the measured value (**A1**) and the change rate (**A2**). (**B**) Analysis results of the intravascular volume of the entire microvasculature: the measured value (**B1**) and the change rate (**B2**). Microvasculatures incubated in VEGF (50 ng/mL)-added medium for 7 days.

Dynamic changes in the entire microvasculature over time were also analysed using OCT images (summarized in Fig. 6). The x−z cross-sectional images were processed with several filters and then converted into binary images to reduce noise from the collagen gel surrounding the microvasculature and give simple black-and-white images of microvasculature lumens. The luminal diameter and intravascular volume were calculated using these processed images (see details in Method section). Under the static culture conditions used in this study, both the luminal diameter (Fig. 6A1) and intravascular volume (Fig. 6B1) were increased with time. After 7 days of incubation, for example, the lumen diameters of each of three microvasculatures were 95.6, 229.2, and 368.4 μm, respectively, increased from 55.9, 153.6, and 261.7 μm on day 0. Interestingly, as shown by the rate of change in Fig. 6A2 and B2, the smaller diameter enlarged. The microvasculature with the smallest diameter (prepared with O.D. 120 μm micro-needles) increased their volume 3.7-fold, whereas those with larger diameters (prepared with O.D. 200 and 300 μm micro-needles) increased only 2.5- and 2.1-fold, respectively. There were no significant changes in the controls incubated without VEGF (see Fig. S2 in the Supplementary Information). These results indicated that VEGF induced proliferation of HUVECs, leading to expansion of the entire microvasculature as well as increased sprouts in the neovasculature. By using a 3D *in vitro* microvasculature model, dynamic changes in an entire system such as response of host vasculature to VEGF stimulation as well as the developing neovasculature over time was successfully detected, capturing physiological phenomena during the angiogenic process which is not demonstrated by the traditional bead assay method.

## Conclusions

In summary, we prepared 3D *in vitro* microvasculatures as a model to view physiologically relevant angiogenesis and were able to carry out live imaging successfully using stage-top OCT. The *in vitro* angiogenic process was clearly and non-invasively monitored with no need for fluorescence staining. The obtained OCT images captured 3D features both of prepared microvasculatures and neovessel sprouts, showing their advantage over BFM. The stage-top system enabled simple, convenient scanning, providing images through the several-millimetre scale area within a short time. Additionally, cross-sectional images obtained at the point of sprouting clearly visualized the luminal formation process, allowing us to measure the dynamic changes in the sprout, including its luminal structures as well as traditional parameters, such as sprout number and length. There are a number of conventional observation techniques to monitor cell growth in live samples without staining. However, quick live scanning of three-dimensional tissues with relatively large volume (i.e., millimeter scale) for a long surveillance period is still challenging. The resolution of OCT is still lower than that of optical microscope systems, limiting it to imaging details of cell structure. However, OCT provides simple, rapid, noninvasive imaging of 3D tissues and promises great utility not only for angiogenesis of blood vessels but other physiological events such as growth of spheroids or luminal formation. Due to rapid growth of the field of 3D *in vitro* models or organs on a chip, simple and versatile detection methods for long culture period such as OCT technique will become increasingly essential for quantitative image analyses.

## Materials and Methods

### Materials

Type I-A Cellmatrix^®^ collagen derived from porcine tendons by acid extraction (3 mg/mL, pH 3) was obtained from Nitta Gelatin Inc. (Osaka, Japan). Acupuncture needles (120-μm diameter: J type, No. 02, 30-mm length; 200-μm diameter: J type, No. 3, 30-mm length; 300-μm diameter: J type, No. 8, 60-mm length) were purchased from Seirin Co. Ltd. (Shizuoka, Japan). Fibronectin solution (1 mg/mL) was purchased from Biomedical Technologies Inc. (Stoughton, MA, USA). Hoechst 33342 and Alexa Fluor^®^ phalloidin 488 were purchased from Thermo Fisher Scientific, Inc. (Waltham, MA, USA). Human vascular endothelial growth factor (VEGF_165_) recombinant, 0.5 w/v% trypsin-5.3 mmol/l EDTA-4Na solution, and 10 × phosphate buffer saline (-) (PBS) were purchased from Wako Pure Chemical Industries, Ltd. (Osaka, Japan). Dextran from *Leuconostoc* spp. (M_r_ 450,000–650,000) and Hanks’ Balanced Salt Solution (HBSS) were purchased from Sigma-Aldrich Co. LLC. (St. Louis, MO, USA).

### Cell Culture

Human umbilical vein endothelial cells (HUVECs) and cell culture medium EGM™-2 Endothelial Cell Growth Medium-2 BulletKit™ were purchased from Lonza Japan Ltd. (Tokyo, Japan). HUVECs were seeded on cell culture polystyrene dishes and incubated at 37 °C with 5% CO_2_. Once confluent, cells were harvested after 3 min incubation with trypsin-EDTA 0.25% and collected by centrifugation (400 × *g*, 3 min) prior to resuspension in the medium. HUVECs passage 4 was used to prepare the microvasculature model.

### Device Preparation

Polydimethylsiloxane (PDMS)-based microdevices with dumbbell-shape chambers having a needle inserting channel (diameter 300 μm) were prepared with slight modification of the method previously reported by Tien *et al*.^10^ and kindly gifted by Dai Nippon Printing Ltd. (Tokyo, Japan). Each channel contained a central rectangular area (4 × 6 × 4 mm: width × length × height) for collagen gel and reservoirs (cylinder dimension; 3 × 2 mm: diameter × height) at both ends.

Briefly, a convex mould was fabricated by a 3D printer to produce the PDMS device. The surface of the convex mould was treated with a release agent. A mixture of PDMS and a curing agent (SILPOT 184; Dow Corning Toray Co., Ltd., Tokyo, Japan) at a ratio of 10:1 (w/w) was cast on the convex mould equipped with micro-needles (diameter 300 μm) and subsequently cured at 37 °C overnight. A concave PDMS device was obtained by peeling it off the convex mould. The PDMS device and glass coverslips were treated with O_2_ plasma (PDC-32G; Harrick Plasma, Ithaca, NY) for 60 s, and immediately bonded at 75 °C for 2 h. The PDMS device was cleaned with O_2_ plasma for 60 s and sterilized by exposure to an ultraviolet lamp under the cell culture hood for 5 min prior to cell culture.

### Microvasculature Preparation

A top glass (6.0 × 20.5 mm: width × length) was placed on the chamber to cover the central rectangular channel. The surface of the chamber in the PDMS device was coated with fibronectin (10 μg/mL) in phosphate-buffered saline (PBS) for 30 min. Acupuncture needles at diameters of 120, 200, and 300 μm were coated with 1% (w/w) bovine serum albumin (BSA) in PBS and then inserted through the channel. The neutralized collagen solution (final concentration 2.4 mg/mL) was added in the chamber and incubated at 37 °C for 30 min. The micro-needles were removed from the device to obtain one-side-open collagen micro-channels. A 5-μL aliquot of the HUVECs’ suspension [0.5 × 10^7^ cells/mL for the 120-μm channel or 1.0 × 10^7^ cells/mL for 200- and 300-μm channels in EGM-2 containing 3% (w/w) dextran] were loaded into the collagen gel channels, and incubated at 37 °C for 15 min to allow their attachment to the inner wall of the collagen gel channel. Subsequently, the device was placed in 35 mm Petri dish to avoid vaporization, 1.5 mL of endothelial cell growth medium (EGM-2) was added to cover the collagen gel channels, and HUVECs were incubated at 37 °C, 5% CO_2_ for 2 days. The added medium to the device (1.5 mL) was changed at 3 and 24 h after preparation.

### Angiogenesis induced by VEGF

After 2 days of pre-culture of the microvasculature, the EGM-2 medium was exchanged, and VEGF_165_ solution in PBS was directly added to medium at a final concentration of 50 ng/mL. The devices were then incubated at 37 °C and 5% CO_2_ for 7 days. The medium containing VEGF_165_ was changed every 48 h. Cell morphology and angiogenesis from the microvasculature were monitored under a phase contrast microscope with lenses of 10 × magnification (Axio Observer D1; Zeiss, Oberkochen, Germany).

### OCT Observation

The spectrum-domain OCT (SD-OCT) system is outlined in Fig. 1B1. The system is equipped with an 850-nm light from a super luminescent diode (SLD) and an objective (N.A. = 0.07). The SLD output is coupled into a single-mode fibre and split at the fibre coupler into the microvasculature sample and reference arms. Reflections from the two arms are combined at the coupler and detected by the spectrometer. The microvasculature was imaged from the bottom surface. To acquire data for each 2D x−z cross-sectional image, a series of longitudinal scans were performed with the optical beam position translated laterally between scans (Fig. 1B2). The data acquisition window was 1 × 2 × 1 mm, and the voxel size was 2.56 × 2.56 × 2.56 μm. The lateral resolution of the image is limited by the beam diameter inside the sample. In our measurements, the beam diameter was 10 μm. The minimum image acquisition time for the images consisting of 780 longitudinal scans shown here was ~5 min.

### Image Analysis

Original images obtained by the OCT system contained noise from the collagen gel surrounding the microvasculature. To reduce this noise, image processing was applied for the collected original OCT stack images using ImageJ image processing software (Version 1.50i; NIH) as follows. The images were subsequently processed with filters (3D maximum filter, 3D Gaussian low-pass filter, 2D FFT band-pass filter, and 3D Gaussian low-pass filter). The images were then converted into binary images so that the cell area is white and all other areas are black. 3D images were constructed using the ImageJ Plugin 3D viewer. Sprout length, lumen length, lumen diameter and intravascular volume of the microvasculature were analysed as described below.

#### Sprout Length and Lumen Length

Total sprout length was defined as the distance between starting point and sprout end point. Lumen length was defined as the distance between starting point and lumen end point. These points were manually selected from the 2D images then each distance was measured. Detail of the measurement image are shown in Fig. 5C. Target neovasculatures were selected as the three most elongated sprouts (total length = 153–242 μm) from microvasculature (O.D. = 120 μm).

#### Lumen Diameter and Intravascular Volume of the Microvasculature

To analyse the luminal diameter of prepared host microvasculature, ‘particle analysis’ (size > 500 pixels^2^) was performed after binary image conversion. The width (x axis direction) of the luminal diameter was measured from each image, and mean luminal diameter of 780 slices was calculated. To analyse the intravascular volume, the area of the lumen was measured from each image, and the volume was calculated by multiplying the measured area × the distance between slices (2.56 μm) for 780 slices (total microvasculature length 2 mm).

### Immunohistochemistry and CLSM Observation

Cells and extracellular matrix were visualized using immunofluorescence staining. Briefly, after washing with PBS, the microvasculature in the PDMS device was fixed with 4% (w/w) paraformaldehyde in PBS for 30 min and then permeabilized with 0.5% Triton-X in PBS for 10 min. Blocking was performed using 1% BSA in PBS overnight at 4 °C. For staining F-actin, the cells were exposed to Alexa Fluor 488-phalloidin at 25 °C for 2 h, and for nucleus, Hoechst 33342 at 25 °C for 15 min. For staining laminin and podocalyxin, after blocking, the microvasculature was incubated with rabbit polyclonal antibody against laminin (1:100; ABcam) and goat polyclonal anti-human podocalyxin (1:100; R&D) overnight at 4 °C. The microvasculature was then washed well with PBS and exposed to Alexa Fluor 568-secondary antibodies and Alexa Fluor 488-phalloidin together. Both anti-rabbit and anti-goat secondary antibodies (Invitrogen) were used at 1:100 dilutions. Images of the stained 3D microvasculature were obtained using a confocal microscope (LSM700; Zeiss, Oberkochen, Germany) with lenses of 10 × magnification. Confocal images were processed using the software ZEN version 8.1 (Zeiss) to construct 3D projection images from Z-stack images.

## Additional Information

**How to cite this article:** Takahashi, H. *et al*. Visualizing dynamics of angiogenic sprouting from a three-dimensional microvasculature model using stage-top optical coherence tomography. *Sci. Rep.* **7**, 42426; doi: 10.1038/srep42426 (2017).

**Publisher's note:** Springer Nature remains neutral with regard to jurisdictional claims in published maps and institutional affiliations.

## Supplementary Material

Supplementary Information

Supplementary Movie S1

Supplementary Movie S2

Supplementary Movie S3

Supplementary Movie S4

Supplementary Movie S5
