# Supplementary material for: Visualizing dynamics of angiogenic sprouting from a three-dimensional microvasculature model using stage-top optical coherence tomography
Source: Sci Rep. 2017 Feb 10;7:42426. doi: 10.1038/srep42426 (PMC5301260; doi:10.1038/srep42426)
Supplement: Supplementary Information [file srep42426-s1.pdf]

Supplementary Information

Visualizing dynamics of angiogenic sprouting from a three-dimensional microvasculature model using stage-top optical coherence tomography

Haruko Takahashi,[1] Keisuke Kato,[2] Kenji Ueyama,[2] Masayoshi Kobayashi,[2] Gunwoong Baik,[1] Yasuhiro Yukawa,[3] Jun-ichi Suehiro[4] & Yukiko T. Matsunaga[1,*]

[1] Center for International Research on Integrative Biomedical Systems, Institute of Industrial Science, The University of Tokyo, 4-6-1 Komaba, Meguro-ku, Tokyo 153-8505, Japan

[2] R&D Department 1, Screen Holdings Co., Ltd., 322 Furukawa-cho, Hazukashi, Fushimi-ku, Kyoto 612-8486, Japan

[3] Centre for Interdisciplinary Research on Micro-Nano Methods, Institute of Industrial Science, The University of Tokyo, 4-6-1 Komaba, Meguro-ku, Tokyo 153-8505, Japan

[4] Department of Pharmacology and Toxicology, Kyorin University School of Medicine, 6-20-2 Shinkawa, Mitaka-shi, Tokyo 181-8611, Japan

* To whom correspondence should be addressed: Yukiko T. Matsunaga

E-mail: mat@iis.u-tokyo.ac.jp

Tel: +81-3-5452-6470

Fax: +81-3-5452-6471

Table of contents.

SI. 1: Figure S1. Evaluation of initial microvasculatures after 2-day pre-incubation.

SI. 2: Figure S2. VEGF-induced angiogenesis.

SI. 3: Caption of Movie S1.

SI. 4: Caption of Movie S2.

SI. 5: Caption of Movie S3.

SI. 6: Caption of Movie S4.

SI. 7: Caption of Movie S5.

* Movies S1−S5 are filed and served individually.

SI. 1: Figure S1. Evaluation of initial microvasculatures after 2-day pre-incubation.

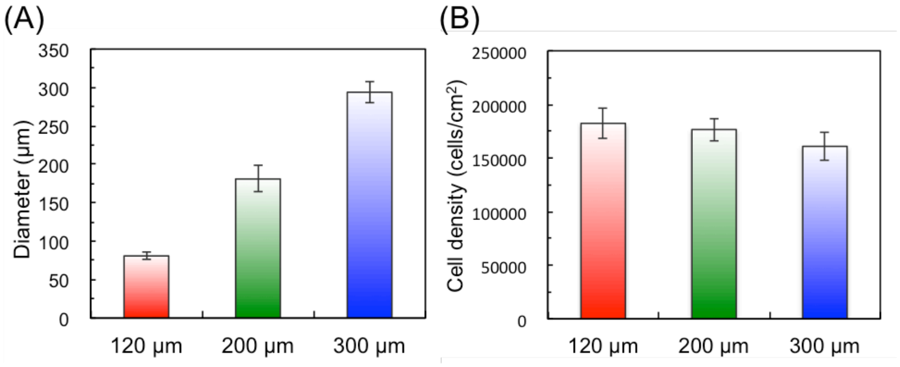

Figure S1. Evaluation of initial microvasculatures after 2-day pre-incubation. (**A**) Outer diameter (O.D.) of 2-day pre-incubated microvasculatures measured using brightfield microscopy. (**B**) Cell density of 2-day pre-incubated microvasculatures. The nuclei of HUVECs were stained using Hoechst 33342 and detected by confocal laser scanning microscopy.

SI. 2: Figure S2. VEGF-induced angiogenesis.

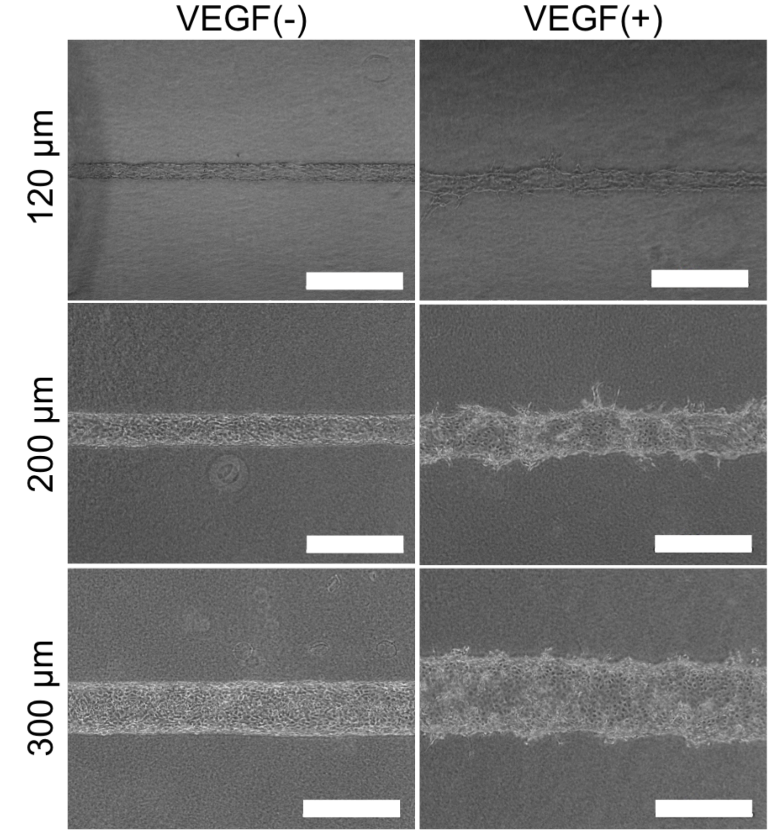

Figure S2. Brightfield microscopy images of vascular endothelial growth factor (VEGF)-induced angiogenesis. These images are taken after 7 days of incubation without additional VEGF stimulation (left) or with VEGF (50 ng/mL) (right). Bars = 500 µm.

SI. 3: Caption of Movie S1.

Movie S1. Dynamic changes in the microvasculature. 3D images of entire microvasculature (2 mm in length) with 200-µm diameter on days 0–7.

SI. 4: Caption of Movie S2.

Movie S2. 3D image of entire microvasculatures (2 mm in length) with 200-µm diameter at 0 day.

SI. 5: Caption of Movie S3.

Movie S3. 3D image of entire microvasculatures (2 mm in length) with 200-µm diameter at 7 days.

SI. 6: Caption of Movie S4.

Movie S4. Dynamic changes in angiogenic sprouting. 3D images of sprouting from the 120-μm diameter microvasculatures on days 0−7.

SI. 7: Caption of Movie S5.

Movie S5. Dynamic changes in angiogenic sprouting. 3D images of sprouting from the 200-μm diameter microvasculatures on days 0−7.
